# Epigenetic remodeling to improve the efficacy of immunotherapy in human glioblastoma: pre-clinical evidence for development of new immunotherapy approaches

**DOI:** 10.1186/s12967-024-05040-x

**Published:** 2024-03-01

**Authors:** Maria Fortunata Lofiego, Francesca Piazzini, Francesca Pia Caruso, Francesco Marzani, Laura Solmonese, Emma Bello, Fabrizio Celesti, Maria Claudia Costa, Teresa Noviello, Roberta Mortarini, Andrea Anichini, Michele Ceccarelli, Sandra Coral, Anna Maria Di Giacomo, Michele Maio, Alessia Covre, The EPigenetic Immune-oncology Consortium Airc (EPICA) investigators

**Affiliations:** 1 University of Siena, Siena, Italy; 2 BIOGEM Institute of Molecular Biology and Genetics, Ariano Irpino, Italy; 3 Department of Electrical Engineering and Information Technology (DIETI), University of Naples “Federico II”, Naples, Italy; 4 Center for Immuno-Oncology, University Hospital of Siena, Siena, Italy; 5 Human Tumors Immunobiology Unit, Department of Research, Fondazione IRCCS Istituto Nazionale dei Tumori, Milan, Italy; 6 Sylvester Comprehensive Cancer Center, Miller School of Medicine, University of Miami, Miami, FL USA; 7 Department of Public Health Sciences, Miller School of Medicine, University of Miami, Miami, FL USA

**Keywords:** Glioblastoma, Brain metastases, Immunotherapy, DNA hypomethylating agent, Melanoma

## Abstract

**Background:**

Glioblastoma multiforme (GBM) is a highly aggressive primary brain tumor, that is refractory to standard treatment and to immunotherapy with immune-checkpoint inhibitors (ICI). Noteworthy, melanoma brain metastases (MM-BM), that share the same niche as GBM, frequently respond to current ICI therapies. Epigenetic modifications regulate GBM cellular proliferation, invasion, and prognosis and may negatively regulate the cross-talk between malignant cells and immune cells in the tumor milieu, likely contributing to limit the efficacy of ICI therapy of GBM. Thus, manipulating the tumor epigenome can be considered a therapeutic opportunity in GBM.

**Methods:**

Microarray transcriptional and methylation profiles, followed by gene set enrichment and IPA analyses, were performed to study the differences in the constitutive expression profiles of GBM vs MM-BM cells, compared to the extracranial MM cells and to investigate the modulatory effects of the DNA hypomethylating agent (DHA) guadecitabine among the different tumor cells. The prognostic relevance of DHA-modulated genes was tested by Cox analysis in a TCGA GBM patients’ cohort.

**Results:**

The most striking differences between GBM and MM-BM cells were found to be the enrichment of biological processes associated with tumor growth, invasion, and extravasation with the inhibition of MHC class II antigen processing/presentation in GBM cells. Treatment with guadecitabine reduced these biological differences, shaping GBM cells towards a more immunogenic phenotype. Indeed, in GBM cells, promoter hypomethylation by guadecitabine led to the up-regulation of genes mainly associated with activation, proliferation, and migration of T and B cells and with MHC class II antigen processing/presentation. Among DHA-modulated genes in GBM, 7.6% showed a significant prognostic relevance. Moreover, a large set of immune-related upstream-regulators (URs) were commonly modulated by DHA in GBM, MM-BM, and MM cells: DHA-activated URs enriched for biological processes mainly involved in the regulation of cytokines and chemokines production, inflammatory response, and in Type I/II/III IFN-mediated signaling; conversely, DHA-inhibited URs were involved in metabolic and proliferative pathways.

**Conclusions:**

Epigenetic remodeling by guadecitabine represents a promising strategy to increase the efficacy of cancer immunotherapy of GBM, supporting the rationale to develop new epigenetic-based immunotherapeutic approaches for the treatment of this still highly deadly disease.

**Supplementary Information:**

The online version contains supplementary material available at 10.1186/s12967-024-05040-x.

## Introduction

Glioblastoma multiforme (GBM) is a highly aggressive and malignant type of primary brain tumor, challenging to treat due to its rapid growth, infiltrative nature, and resistance to conventional therapies [1]. GBMs are astrocytomas of grade IV, which originate from the glial cells that provide support and nourishment to the neurons in the brain. These tumors are typically characterized by the ability to infiltrate nearby healthy brain tissue, making complete surgical removal challenging and often leading to tumor recurrence [2]. Standard treatment for GBM usually involves a combination of surgical resection, followed by concomitant radiation and chemotherapy. However, despite aggressive treatment, GBM often recur, and the prognosis for patients is generally poor. The median survival time after diagnosis is relatively short (15 months), with the worst 5-year life expectancy among all human cancers (less than 7%), emphasizing the need for continued research into more effective treatment strategies [3].

Immunotherapy using immune checkpoint inhibitors (ICI) holds great promise for cancer treatment, becoming increasingly used in the treatment of different types of solid cancers and demonstrating efficacy in lung cancer and melanoma patients with brain metastases (BM) [4–7]. Despite the considerable number of ongoing clinical investigations, ICI is not as effective as would be desired for GBM treatment. Indeed, the phase III CheckMate 498 (NCT02617589) and 548 (NCT02667587) trials have failed to meet their primary endpoint of improving the median overall survival (OS) [8, 9]. Despite these disappointing clinical results, the anti-PD-1 mAb pembrolizumab, given in a neoadjuvant setting to patients with recurrent GBM, significantly improved OS and progression-free survival and demonstrated a functional activation of tumor-infiltrating lymphocytes and a production of an interferon-gamma (IFN-γ) response within the tumor microenvironment (TME) [10].

The lack of clinically significant responses to ICI in GBM can be attributed to the immune environment of the brain, which includes the presence of brain-resident microglia, and the blood–brain barrier (BBB) that controls inflammation and maintains neural function [11–13]. However, the clinical failures of ICI therapy in GBM cannot be entirely attributed to the immune specialization of the brain, as patients with BM from extracranial primary tumors showed responses to ICI. Beyond tumor cell-intrinsic effects (e.g., type of driver mutations), several pronounced differences between GBM and BM have been discovered, demonstrating an abundance of tumor-associated macrophages, tissue-resident microglia, and monocyte-derived macrophages recruited from the peripheral circulation, and a paucity of tumor-infiltrating CD3 + lymphocytes and of immune checkpoints expression [14] in GBM compared to BM [15]. Thus, tumors that arise within the brain actually shape their TME differently than tumors that metastasize to the brain from extracranial sites.

New treatment concepts, targeting different arms of the cancer-immune equilibrium to enhance the clinical response against GBM cells, have been explored in recent years. These include the target of epigenetic derangements that are known to contribute to the pathogenesis of glioma and to its highly immunosuppressive microenvironment, downregulating the tumor expression of different immune molecules, including major histocompatibility complex (MHC) class I and class II proteins, affecting antigen presentation, and of STING, with a subsequent effect on IFN-γ production and cytokine/chemokine release [16]. Recent studies described the impact of epigenetic intervention to reprogram the immune TME toward more antitumoral activity. Among these, increased expression of immunogenic antigens, induced by the DNA hypomethylating agent (DHA) decitabine, increases the ability for antigen-specific cytotoxic T lymphocytes (CTL) to recognize and kill GBM cells [17]. Along this line, it has been highly demonstrated that epigenetic remodeling of cancer cells of different histotypes by decitabine and guadecitabine induced/up-regulated the expression of different immune molecules (i.e., HLA class I, cancer-testis antigens (CTA), co-stimulatory molecules, interferon-stimulated genes), resulting in improved immune recognition of tumor cells [18–22]. Noteworthy, the potential of DHAs to cross the BBB to reach tumor cells within the brain were confirmed by several in vivo studies [23, 24].

Starting from this evidence, this work foresaw to shed light on the epigenetics involvement in similarities and differences of the biology and immune-related transcriptional programs in tumor cells belonging to primary and metastatic brain malignancies. To this end, we carried out a comparative gene expression and methylation profiling of GBM vs melanoma (MM)-BM cell lines to identify their gene signature constitutively differentially expressed, their modification upon DHA treatment and more in general the role of DHA to increase GBM immunogenicity. Results showed that the most striking differences between GBM vs MM-BM cell lines rely on the enrichment of biological themes associated with tumor growth, invasion, and extravasation and to inhibition of antigen processing and presentation in the context of MHC class II in GBM cells. A high number of these differences were associated with the expression of genes regulated by promoter methylation. Accordingly, treatment with DHA activated several genes, constitutively down regulated in GBM vs MM-BM, related to the activation, proliferation, and migration of T and B cells and activation of the antigen processing and presentation via MHC class II molecules. This demonstrates that epigenetic mechanisms mediate the reprogramming of GBM cells, bringing them closer to the more immunogenic phenotype observed in MM-BM cells. In general, guadecitabine treatment activated several transcriptional factors related to innate and adaptative immune responses both on primary and secondary brain tumors.

Noteworthy, a set of genes modulated by guadecitabine in GBM cells significantly correlated with a prognostic role in the cohort of GBM patients from The Cancer Genome Atlas Program (TCGA) database.

Stemming from this data, our results support the rationale to develop epigenetically-based immunotherapeutic approaches to enhance brain malignancies patients’ clinical outcome and quality of life, especially for GBM patients.

## Materials and methods

### Tumor cell lines

GBM (n = 14) and MM-BM (n = 12) cell lines were established from surgically removed tumor tissues of GBM and MM-BM patients undergoing surgery at the Department of Neurosurgery of University Hospital of Siena, under approval by the Committee on Human Research. Tumor tissues were processed within 60 min following surgical removal and were dissected into fragments by mechanical digestion (1–2 mm^3^) washed with PBS 1X and cultured, according to the size in T25 cm^2^ or T75 cm^2^ tissue culture flasks. Cells were cultured using RPMI Medium 1640 (Biochrom, Berlin, Germany), supplemented with 20% heat-inactivated fetal bovine serum (FBS, Biochrom, Berlin, Germany), 2mML-glutamine and 100 µg/µl penicillin/streptomycin (Biochrom, Berlin, Germany) up to the 5th step of culture and with 10% heat-inactivated FBS for subsequent ones. MM cell lines (n = 11), kindly provided by Dr. Roberta Mortarini (Human Tumors Immunobiology Unit, Department of Research, Fondazione IRCCS Istituto Nazionale dei Tumori, Milan, Italy) were established as previously described [25]. Origin of cell lines and their characteristics are described in Additional file 1: Tables S1–S3.

### In vitro treatment of GBM, MM-BM and MM cells with guadecitabine

Treatment with guadecitabine was performed as previously described [18]. Briefly, GBM, MM-BM and MM cell lines were seeded in T75 cm^2^ tissue culture flasks (day 0), treated with 1 µM guadecitabine every 12 h for 2 days (day 1, day 2), and harvested at day 5. Control cells were treated under similar experimental conditions in the absence of drug. Guadecitabine was supplied by MedChemExpress LLC (Monmouth Junction, NJ, USA).

### Gene expression profiling

Total RNA was extracted from investigated tumor cell lines using Trizol reagent (Invitrogen, Milan, Italy) according to the manufacturer’s instruction. RNA extracted was digested with RNAse-free DNAse (Roche Diagnostics, Milan, Italy) and 500 ng was subjected to whole transcriptome expression profiling using the Clariom™ S Affymetrix human microarray platform (Affymetrix, Santa Clara, CA). A single GeneChip® Clariom™ S Affymetrix human microarray was then hybridized with each biotin-labelled sense target. Following hybridization, the microarrays were washed and stained with Streptavidin Phycoerythrin on the Affymetrix Fluidics Station 450. Affymetrix GeneChip® Command Console software (Thermo Fisher Scientific, Inc) was used to acquire GeneChip® images and generate.DAT and.CEL files. Gene expression data were analyzed by Transcriptomic Analysis Console software (Applied Biosystems, Thermo Fisher Scientific).

### Genome-wide DNA methylation analysis

Genomic DNA was extracted from investigated cell lines, using QIAmp DNA Blood mini-Kit (Qiagen, Hilden, Germany). DNA methylation analysis was performed on 500 ng of DNA using Methylation EPIC BeadChip (Illumina, San Diego, CA, USA), which contains 850,000 probes. In brief, bisulfite-converted DNA was whole-genome amplified for 20 h followed by end-point fragmentation. Fragmented DNA was precipitated, denaturated, and hybridized to the BeadChips for 20 h at 48 °C. The BeadChips were washed, and the hybridized primers were extended and labeled before scanning the BeadChips using the Illumina iScan system. All samples were randomly placed on each array. The Infinium design is based on specific probes interrogating each CpG site, the signal intensity emitted by their interaction is then measured to generate beta values (β), defined as β = M/(M + U + 100), where M is the intensity corresponding to methylated and U to unmethylated, representing the relative degree of methylation at a locus. The beta values range between 0 and 1, representing fully unmethylated and methylated states, respectively. Raw intensities were processed using minfi R package (v 1.42.0) [26]. Probes with a detected p-value less than 0.05 or mapping short nucleotide polymorphisms (SNPs) were filtered out. Data were normalized according to the Funnorm normalization function [27].

### Data analysis

Identification of differentially (p-value < 0.05) expressed genes (DEGs) among untreated GBM, MM-BM and MM tumor cell types and between treated and untreated cells was performed by BRB-Array Tools package (v4.6.0 Beta 1; https://brb.nci.nih.gov/BRB-ArrayTools/download.html). A p-value of univariate tests < 0.05, p-value threshold for pairwise differences < 0.05 and a gene-level false discovery rate < 0.05 were set as the cut-off criteria analysis. Venn diagrams were generated by InteractiVenn web software [28]. Ingenuity pathway analysis (IPA) comparison was performed on DEGs modulated by DHA treatment in GBM, MM-BM and MM cell lines to identify canonical pathways (CP) and upstream regulators (UR) activated (Z-score ≥ 2) or inhibited (Z-score ≤ − 2) by treatment. Enrichment of Gene Ontology (GO) terms, considering biological process (BPs) was conducted utilizing the EnrichR web-tool [29]. Significative (p-value < 0.05) BPs were ranked based on their combined score value that multiplies the log of the p-value computed with the Fisher exact test by the Z-score computed by our correction to the test and top 50 were analyzed. A univariate Cox proportional hazard model was employed to identify the prognosis-associated DEGs satisfying the criteria of hazard ratio (HR) > 1 or HR < 1 and p-value < 0.05 in TCGA tumor GBM datasets. Scatter plots, violin plots, forest plot and statistical analyses were carried out by GraphPad Prism 8.0 (GraphPad Software Inc., San Diego, CA, USA). A two-tailed paired Student t-test and one-way ordinary ANOVA test were used to calculate p-value for molecular data. p-value < 0.05 was considered statistically significant.

## Results

### Comparative gene expression landscape among GBM, MM-BM and MM cell lines

**Fig. 1:**
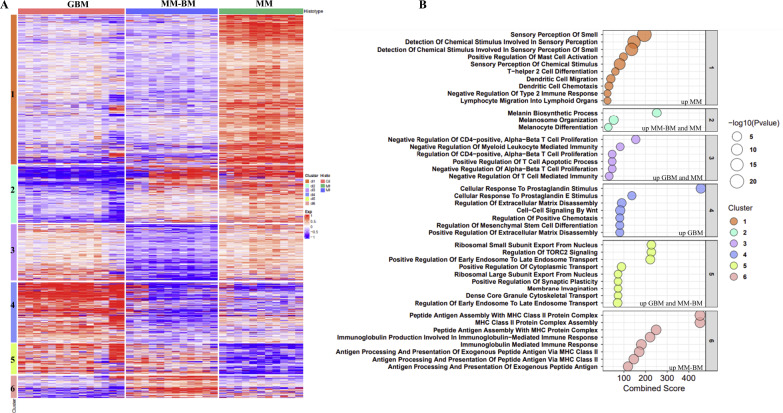
Hierarchical clustering and enrichment analyses of the most variable probes among GBM, MM-BM, and MM cells. **A** Heatmap of Log2 constitutive values of 11,112 DEG (*p*-value < 0.05) clustered according to Euclidean distance between data from whole genome profiling of GBM (#14), MM-BM (#12) and MM (#11) cell lines. **B** Gene Set Enrichment Analysis of genes belonging to each cluster performed by EnrichR tool. X axis reports the Combined Score of significant enriched GO terms. Size of the dot represents the significance of GO terms; colour of the dot represents the different cluster identified by the clustering technique

A total of 14 GBM, 12 MM-BM and 11 MM cell lines, were characterized for their tissue of origin (see Additional file 1: Table S1-S3) and for the whole genome expression profile by Human Clariom™ S arrays. Gene profiling data were analyzed and 11,112 DEGs (*p*-value < 0.05), among the 3 investigated groups of tumor cell types, were identified (see Fig. 1A). These DEGs were analysed by clustering technique to discover distinct patterns of genes: (i) exclusive of each tumor cell type (cluster #1 MM, cluster #4 GBM, cluster #6 MM-BM up-regulated; cluster #2 GBM, cluster #3 MM-BM, cluster #5, MM down-regulated), (ii) influenced by microenvironment and therefore shared between GBM and MM-BM compared to MM (cluster #5 up-regulated; cluster #1 down-regulated), or (iii) pathology-specific and therefore associated with MM and MM-BM compared to GBM (cluster #2 up-regulated; cluster #4 down-regulated) (see Fig. 1A, Additional file 2: Table S1–S12).

**Fig. 2:**
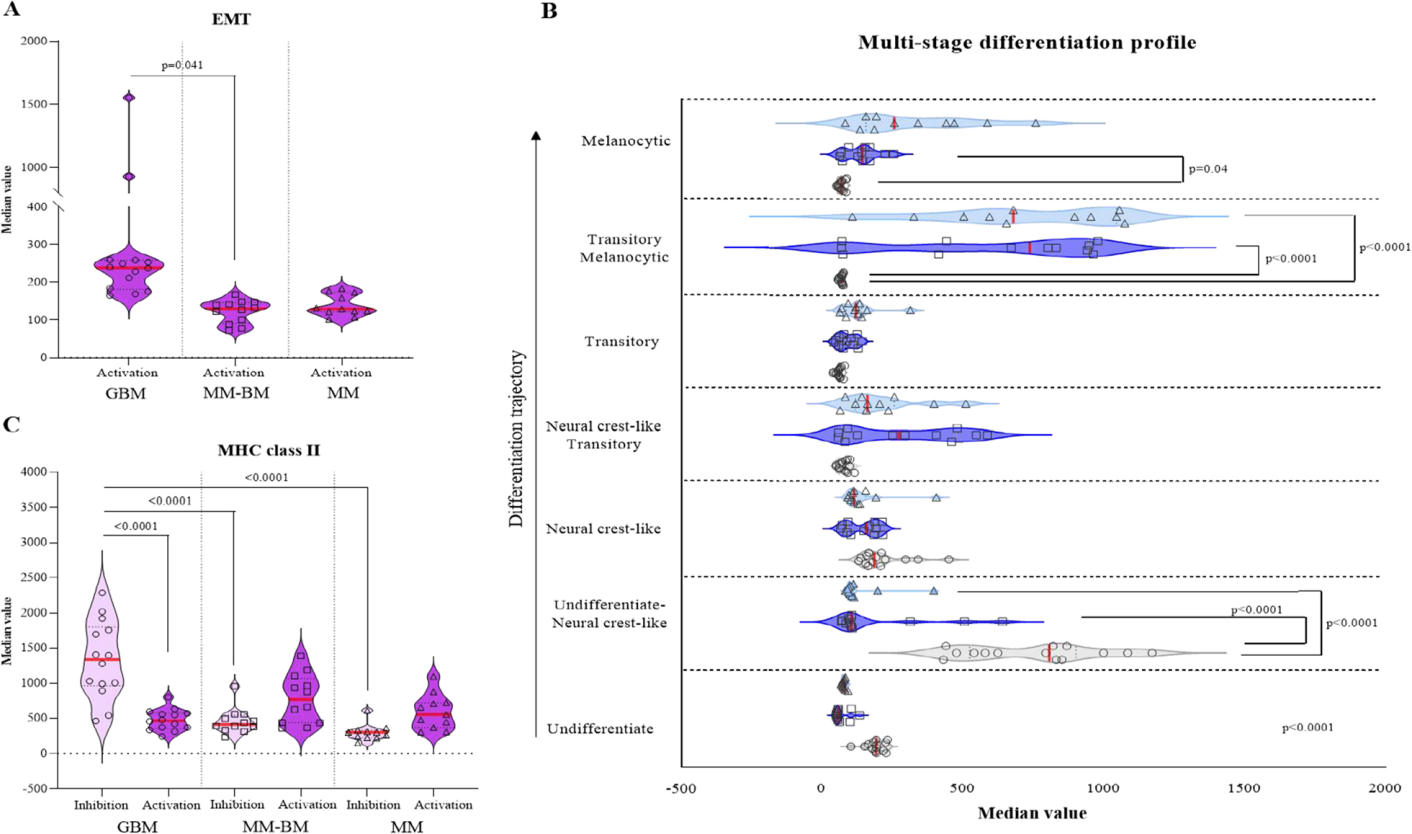
Analysis of specific gene-signatures in GBM, MM-BM and MM cell lines. Starting from whole genome profiling data of GBM (#14), MM-BM (#12) and MM (#11) cell lines, violin plots report the median value of genes expression related to EMT process (#62 genes) modified from MSigDB-GSEA [30] **A**, multi-stage differentiation profile (#517 genes; GBM:grey; MM-BM:blue; MM:light blue) **B**, or to inhibition (#14 genes) or activation (#45 genes) of MHC class II antigen processing/presentation **C** for each tumor cell type. Red line: median value as center; lower and upper dotted lines: 25th and the 75th percentiles, respectively. Statistical significance (p-value < 0.05) assessed by ordinary one-way ANOVA

Comprehensively, the most striking differences between the three groups of tumor cell lines were detected in GBM *vs* MM-BM and/or MM and rely in the activation of biological processes involved in extravasation (cluster #5), chemotaxis (cluster #5), proliferation (cluster #4), immune suppression (cluster #1, cluster #3, cluster #6) and in the negative regulation of antigen processing and presentation in the context of MHC class II (cluster #6). Indeed, genes that were upregulated in GBM *vs* MM-BM enriched for the biological processes related to: (i) a negative regulation of CD4 T cell proliferation and mediated immunity (cluster #3); (ii) a positive regulation of extracellular matrix disassembly (cluster #4) that can influence and regulate the epithelial mesenchymal transition (EMT) process by altering cell–cell and cell-extracellular matrix interactions; (iii) a cellular response to prostaglandin, known to gain an immune suppressive privilege to GBM (cluster #4) (see Fig. 1B, Additional file 2: Table S7, S8). A specific analysis performed on genes related to the activation of the EMT process confirmed an over representation of this signature in GBM compared to MM-BM and MM (see Fig. 2A, Additional file 3: Table S3). In this context, we also explored the expression of the differentiation trajectory-related signatures observing a higher expression of undifferentiated/neural crest-like profiles in GBM cell lines as compared to MM-BM and MM ones (p < 0.0001) (see Fig. 2B, Additional file 3: Table S4). By contrast, MM-BM and MM cell lines were characterized by a higher expression of neural crest-like transitory and of melanocytes specific profiles (see Fig. 2B, Additional file 3: Table S4). Moreover, genes that were down-regulated in GBM *vs* MM-BM and/or MM enriched for the biological process involved in active immune related pathways (cluster #1 and #6), with a particular focus in processes mainly related to MHC class II antigen processing/presentation (cluster #6) (see Fig. 1B, Additional file 2: Table S11, S12). In particular, a down- and an up-regulation of genes involved respectively in the activation and the inhibition of MHC class II antigen processing/presentation, was observed in GBM compared to MM-BM and MM (Fig. 2C, Additional file 3: Table S1, S2).

In addition, through enrichment analysis of genes classified as potentially influenced by “microenvironment” and therefore differentially expressed in primary (GBM) and secondary (MM-BM) brain tumors compared to MM (clusters #1 and #5), it’s noteworthy that the switching off in brain tumors of biological functions related to: perception of smell, mainly driven by the expression of several olfactory receptors, known to be overexpressed in a variety of cancers, including melanoma, that induced inflammasome activation via IL-1 pathway [31]; Type 2 immune response; dendritic cell maturation (cluster #1) (see Fig. 1B, Additional file 2: Table S1, S2). By contrast, genes switched on in brain tumors (cluster #5) enriched for biological processes involved in tumorigenesis and dissemination of tumor cells (see Fig. 1B, Additional file 2: Table S9, S10).

As expected, biological processes related to melanoma biosynthesis and melanocytes differentiation, were overrepresented in MM and MM-BM compared to GBM (cluster #2) (see Fig. 1B, Additional file 2: Table S3, S4).

Lastly, the expression of GBM-specific genes, randomly selected from GBM tissues literature data, in our investigated GBM cell lines, confirmed that they represent a reliable approach/platform to mimic GBM tumors (see Additional file 4: Table S1).

### Transcriptional landscape of GBM *vs* MM-BM and MM cell lines modulated by DHA treatment

**Fig. 3:**
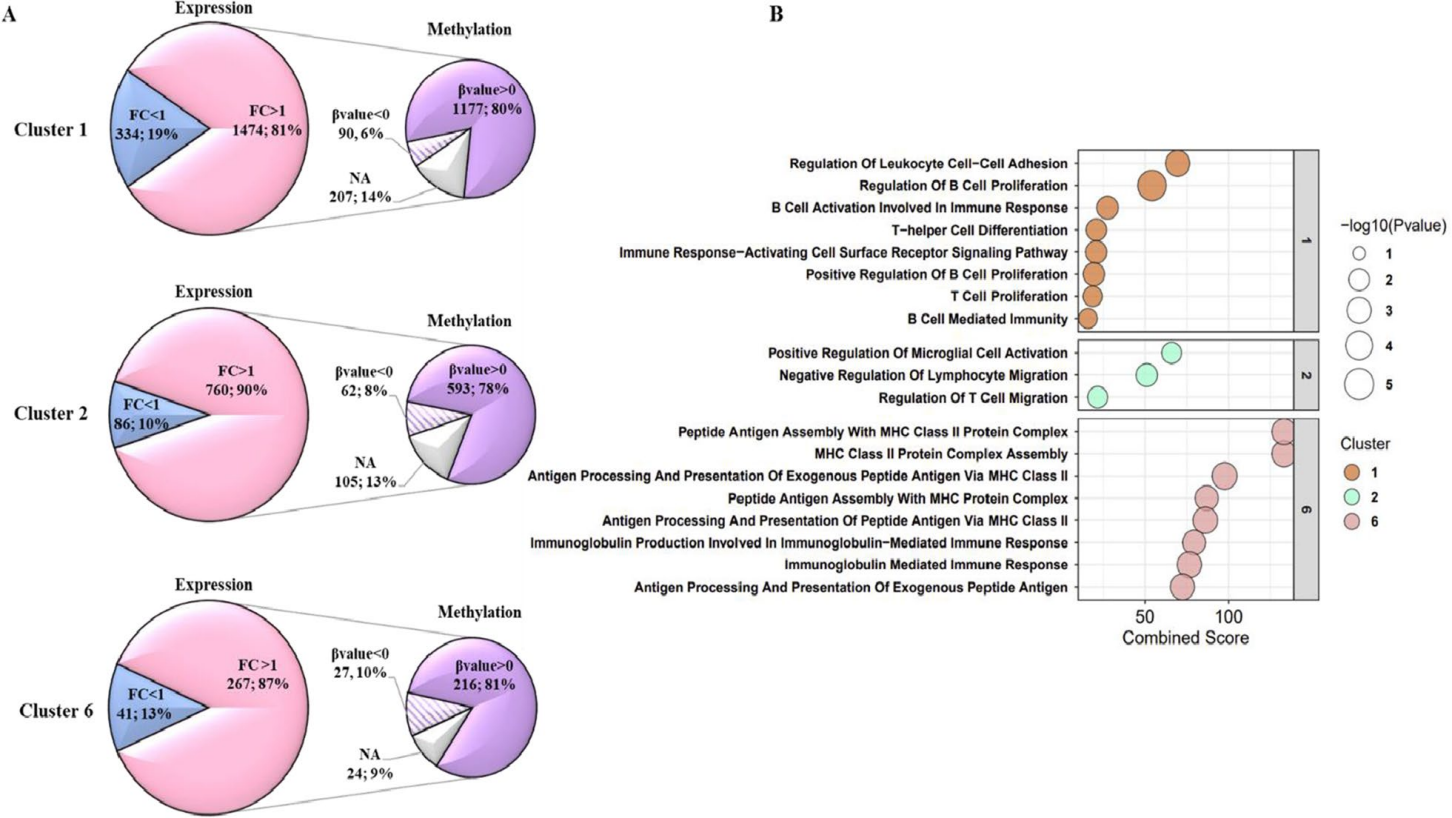
Modulation of DEGs in GBM cells by guadecitabine treatment. **A** Pie of pie charts showed number and percentage of genes belonging to clusters #1, #2, and #6 (see Fig. 1) that were significantly (*p*-value < 0.05) up-regulated (FC > 1) or down-regulated (FC < 1) in DHA-treated *vs* untreated GBM cell lines (expression), and number and percentage of up-regulated genes with ipo- (βvalue < 0) or iper- (βvalue > 0) methylated promoter regions after DHA treatment (methylation, FC βvalue). N/A, genes with no information on the methylation level of their promoter. **B** Enrichment analyses of genes up-regulated by DHA were performed by EnrichR tool. X axis reports the Combined Score of significant enriched GO terms. Size of the dot represents the significance of GO terms; colour of the dot represents the different clusters identified by the clustering technique depicted in Fig. 1

Stemming from the results obtained, in which specific negative immune pathways, in particular the negative regulation of MHC class II antigen processing/presentation and the negative regulation of CD4 T cell proliferation, are enriched in GBM compared to MM-BM, and from the well-known immunomodulatory properties of DHA, we tested the hypothesis that epigenetic remodeling of GBM cells could render them phenotypically closer to the more immunogenic MM-BM and MM cells. To this end, the whole genome expression profile of DHA-treated GBM, MM, and MM-BM cell lines was characterized by Clariom S arrays. We mainly focused on changes induced by the DHA in the expression of DEG constitutively down-regulated in GBM (clusters #1, #2 and #6), and therefore potentially silenced by DNA methylation. Results demonstrated that among genes belonging to these clusters and significantly (*p*-value < 0.05) modulated by DHA-treatment, 81%, 90% and 87% were up-regulated (FC > 1) in clusters #1, #2, and #6, respectively (see Fig. 3A). Intriguingly, biological processes enriched by these up-regulated genes mainly associated with immune-related pathways involved in the activation, proliferation and migration of T and B cells and in the antigen processing and presentation via MHC class II (Fig. 3B). Interestingly, DNA methylation profiles, obtained for each investigated tumor cell lines, revealed a median reduction of methylation levels specifically detected in the promoter regions of 69%, 76% and 78% of the DHA-up-regulated genes belonging to clusters #1, #2, and #6, respectively, in GBM. (Fig. 3A). These results support the direct involvement of methylation in modeling most of the phenomena, including immune suppressive processes, enriched in GBM *vs* MM-BM or MM, previously observed (Fig. 1).

**Fig. 4:**
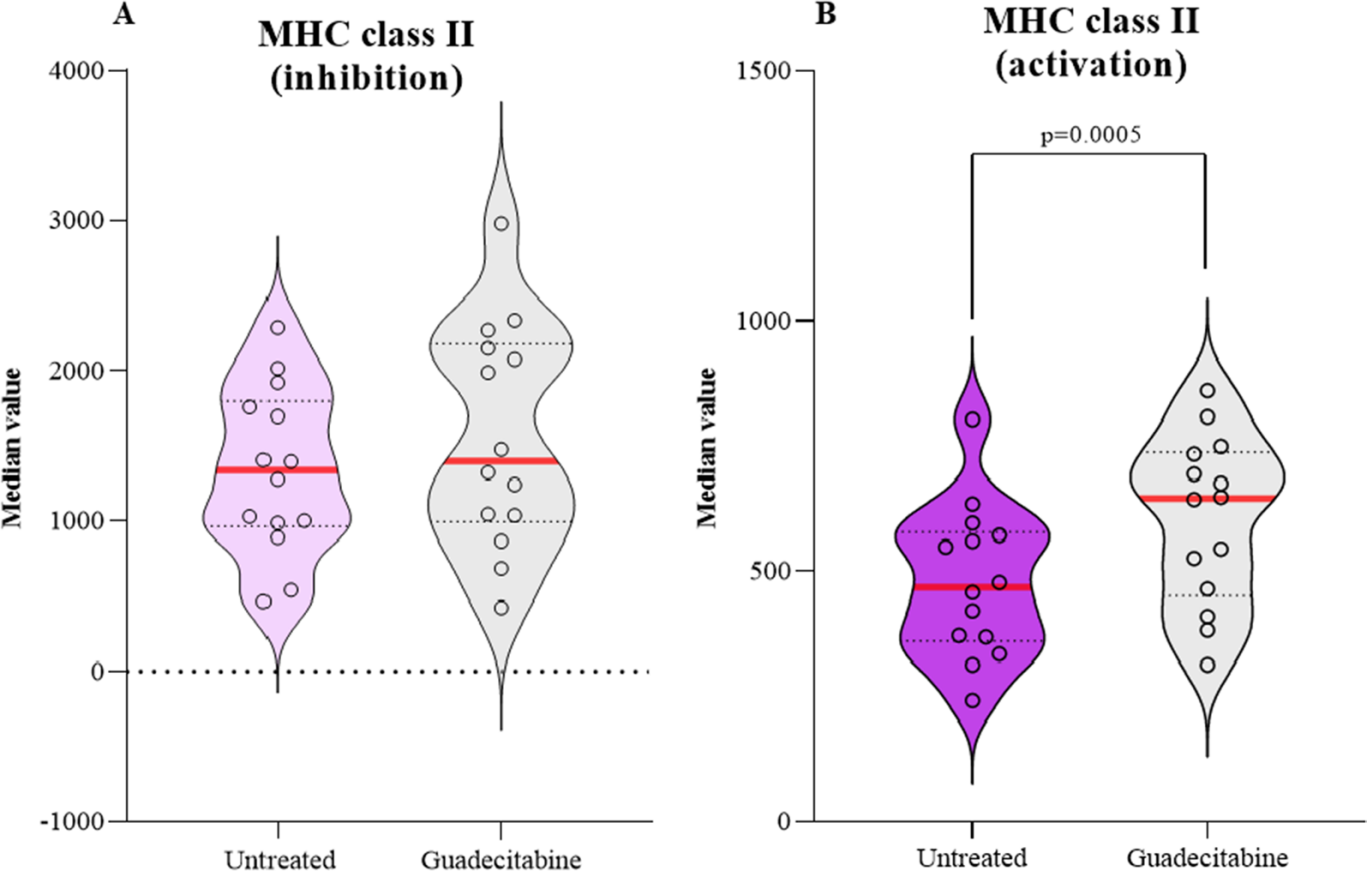
Modulation of gene expression signatures related to MHC class II pathways by DHA, in GBM cell lines. Starting from whole genome profiling data of untreated and DHA-treated GBM (#14) cell lines, violin plots report the median value of the expression of genes related to the A) activation (#45) or B) inhibition (#14) of MHC class II antigen processing/presentation in each DHA-treated and untreated GBM cell lines. Red line: median value as center; lower and upper dotted lines: 25th and the 75th percentiles, respectively. Statistical significance (p-value < 0.05) assessed by two-tailed paired t test

Results from these enrichment analyses were corroborated by changes in the expression of specific genes, selected for their function in activating or inhibiting MHC class II antigen processing/presentation, observed in GBM cell lines after DHA treatment (see Fig. 4, Additional file 3: Table S1). In detail, a statistically significant (p = 0.0005) up-regulation (median FC = 1.4) in the expression of activating genes was observed in DHA-treated *vs* untreated GBM cell lines; whereas expression of genes negatively-regulating MHC class II antigen processing/presentation was not affected by the treatment (see Fig. 4, Additional file 3: Table S2).

### Biological function of genes modulated in GBM, MM-BM and/or MM cell lines, by DHA treatment

**Fig. 5:**
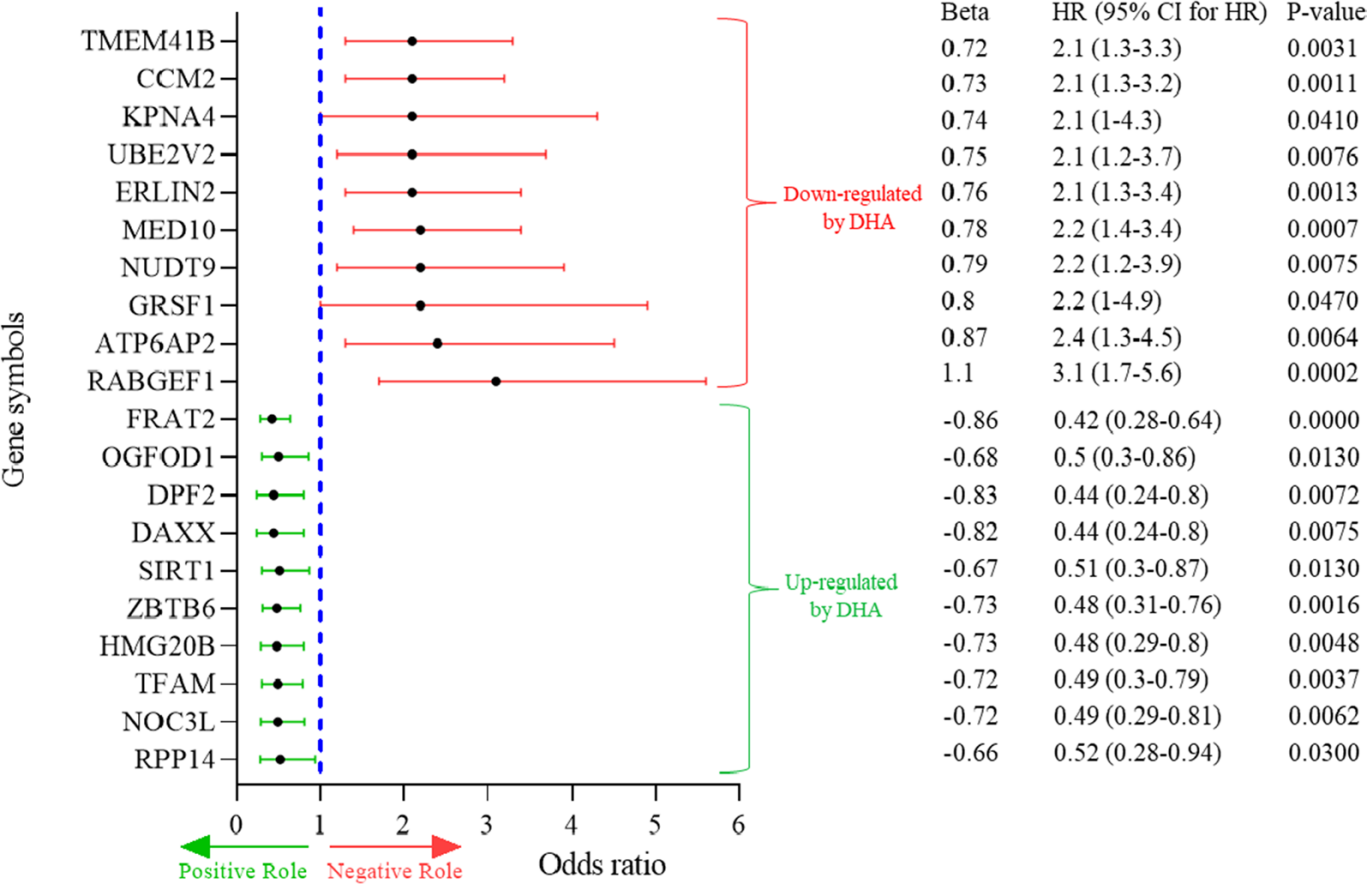
Prognostic role of DEGs modulated by guadecitabine in GBM. Forest plot showed the top 10 genes with the highest positive or negative influence on TCGA GBM patients' OS screened by univariate Cox regression analysis (*p*-value < 0.05). Each gene is accompanied by a point estimate of its hazard ratio and 95% confidence interval

To study the global effects of DHA treatment, analysis of the whole genome expression profile in guadecitabine-treated *vs* untreated tumor cell lines was further conducted. Results identified a total of 6531, 2594 and 2668 DEGs, significantly (*p*-value < 0.05) modulated in GBM, MM-BM or MM treated *vs* untreated cell lines, respectively (see Additional file 8: Fig. S1). A univariate Cox regression analysis was performed on the 6531 DHA-modulated GBM-specific DEGs to assess their potential function as significant predictors of prognosis for GBM patients (n = 154) within the TCGA dataset. This model identified a total of 496 DHA-modulated genes significantly correlated with a prognostic role in the cohort of GBM patients (see Additional file 5: Table S1). Interestingly, the top 10 genes with the highest positive or negative prognostic role were up- and down-regulated, respectively, by guadecitabine (see Fig. 5, Additional file 5: Table S2).

To study the biological activity of DHA treatment on investigated tumor cell types, a comparison analysis of canonical pathways (CPs) was performed on the identified DHA-modulated DEGs, by IPA. GBM cell lines exhibited the highest number (#139) of univocal CPs predicted to be modulated by DHA, compared to MM-BM (#11) and MM (#9) cells (see Additional file 6: Table S1). Among those specifically modulated in GBM, only 3 CPs were predicted to be inhibited by DHA (i.e., IL-10 Signaling, Antioxidant Action of Vitamin C, PPAR Signaling) whereas 136 were activated. These activated CPs were involved in gliomagenesis, neuronal and neurotransmission cellular process and, more importantly, in the activation of immune response (see Additional file 6: Table S1).

**Fig. 6:**
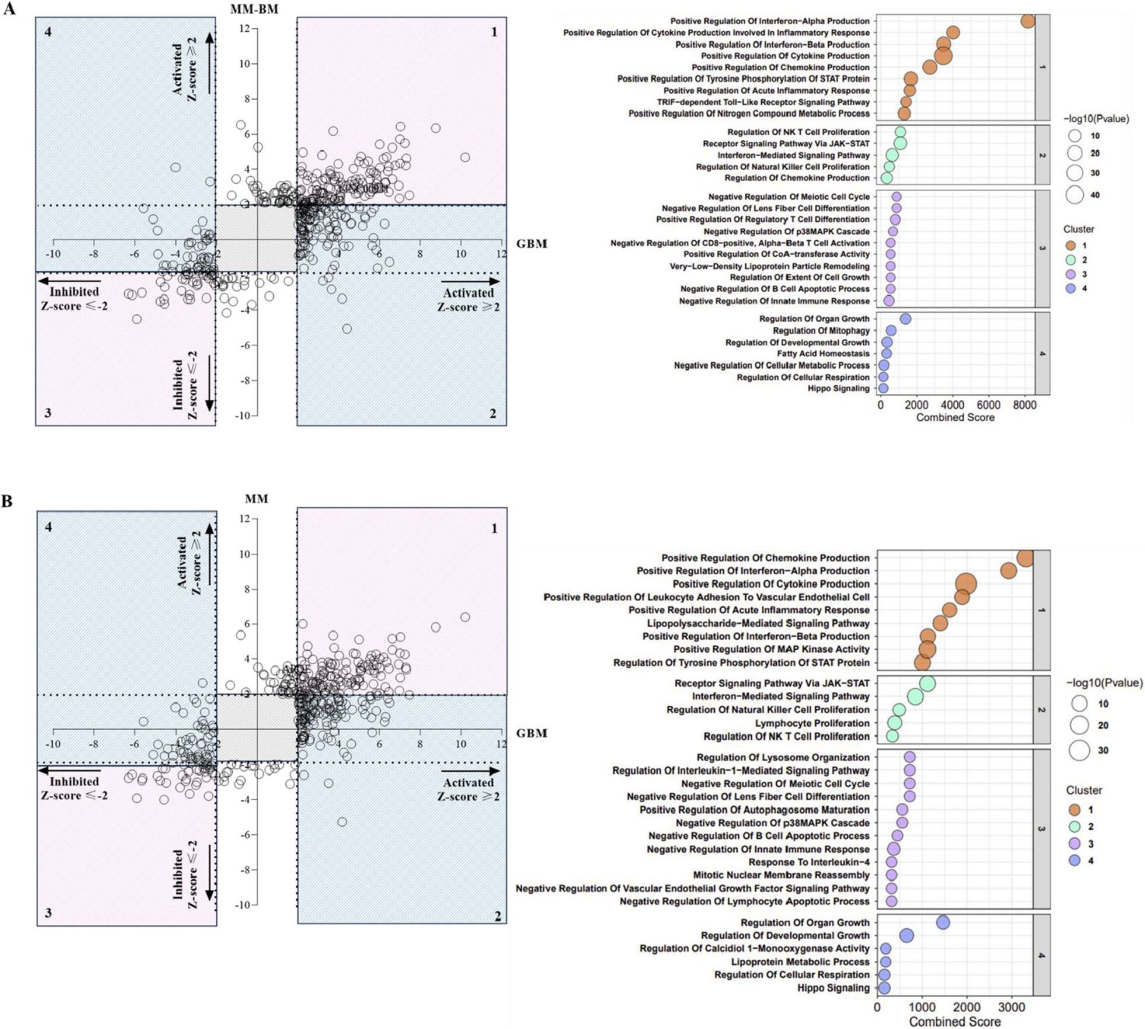
Scatter plot of representative biological processes enriched by URs modulated by DHA treatment. Starting from whole genome profiling data of untreated and DHA-treated GBM (#14), MM-BM (#12) and MM (#11) cell lines, comparative IPA analyses of biological processes enriched by URs was performed. Pink squares represent biological processes enriched by URs significantly activated (#1) or inhibited (#3) after guadecitabine treatment and shared by **A** GBM and MM-BM and/or by **B** GBM and MM cell lines. Light blue squares represent biological processes enriched by URs significantly activated (#2) or inhibited (#4) after guadecitabine treatment exclusively in **A** GBM compared to MM-BM or in **B** GBM compared to MM cell lines. The grey squares represent area of no-significance for Z-score and *p*-value. Gene Set Enrichment Analysis of genes belonging to each cluster was performed by EnrichR tool. Representative biological processes among the top 50 are listed in the scatter plot: X axis reports the Combined Score of significant enriched GO terms; size of the dot represents the significance of GO terms; colour of the dot represents the different cluster identified by the clustering technique

To strengthen the comparative characterization of the effect of guadecitabine in GBM *vs* MM-BM and/or MM cell lines, we carried out further comparison IPA analyses by looking at upstream regulators (URs) that specifically control the observed gene expression changes, in each tumor cell type (see Additional file 7: Table S1). According to comparison analysis, it’s noteworthy that in GBM *vs* MM-BM and/or MM cell lines, guadecitabine treatment modulated URs with an impact on processes involved in immune recognition, by enhancing cellular responses to Type I-, II-, and III IFNs, promoting the activation and proliferation of NK and NK T cells (see Fig. 6, panels #2 A, B; Additional file 7: Tables S2, S4), and shut down URs critically involved in cell proliferation/cell cycle regulation, cellular growth, tissue homeostasis and metabolism. In particular, DHA-inhibited URs in GBM cells were involved in the Hippo signaling pathway, which provides a growth advantage to cancer cells, promoting cancer metastasis, resistance to chemo- and radiotherapy, and a dysregulation of metabolic processes (see Fig. 6 panel #4 A, B; Additional file 7: Tables S3, S5).

In addition to those specifically regulated in GBM, URs activated by DHA and shared between GBM and MM-BM cell lines and/or between GBM and MM cell lines revealed a large set of immune-related activated URs (e.g., cytokines, IFNs, JAK-STAT, Toll-like receptor, and TNF-α superfamily) (see Fig. 6, panels #1 A, B; Additional file 7: Tables S6, S7). These URs enriched for biological processes involved in the regulation of cytokines and chemokines production, inflammatory response and, as observed for biological processes enriched by URs exclusive for GBM cell lines, in Type I, II, III IFN- mediated signaling (see Fig. 6 A, B panels #1; Additional file 7: Tables S6, S7).

Conversely, biological processes enriched by URs commonly inhibited in GBM and MM-BM cell lines and/or in GBM and MM cell lines were mainly involved in metabolic and proliferative pathways, with both positive and negative activity, hindering a conclusive definition of their antitumoral effects and suggesting for more in-depth analyses to better characterize their specific biological functions (see Fig. 6 A, B panels #3; Additional file 7: Tables S8, S9).

## Discussion

The use of ICI has revolutionized modern cancer treatment, generating significant interest in their potential application within neuro-oncology. While some subsets of patients with BMs, associated with a wide range of OS, have exhibited promising and durable responses to immunotherapy [5–7], the efficacy of these treatments has not been as pronounced for the majority of GBM patients [32, 33]. The limited success of immunotherapy in GBM can be attributed to various factors, including the diversity of tumor phenotypes associated with differences in the immune cell composition and functionality within these highly aggressive brain tumors. Indeed, although ICI may initially strengthen T cell function [34] the prevalence of immunosuppressive cells in GBM microenvironment remains a dominant source of resistance to treatment [35]. In light of these considerations, it is crucial to explore new strategies that enhance treatment response and improve the survival prospects and quality of life for GBM patients. Recent evidence has highlighted biological differences between primary and secondary brain tumors, emphasizing variations in tumor-infiltrating lymphocytes, the absence of immune checkpoint expression, and lower tumor mutational burden in patients with GBM compared to BM [14]. However, in-depth molecular analyses of the intrinsic characteristics of tumors derived from GBM and BM are lacking. To address this gap, a comparative transcriptomic analysis was performed on the constitutive expression profiles of GBM vs MM-BM, compared to the extracranial MM cell lines. This analysis identified the activation of biological processes involved in extravasation, chemotaxis, proliferation, immune suppression, and in the negative regulation of antigen processing and presentation in the context of MHC class II as the most remarkable differences between GBM vs MM-BM and/or MM. Notably, higher expression of mesenchymal molecules and EMT-regulating markers in GBM compared to MM-BM or MM, are associated with a less favorable outcome in patients with cancer [36, 37] and are an interesting clue to support the disassembly of focal adhesions, capable of inducing migration and invasion that can increase the resistance of GBM cells to multiple treatments strategies. Furthermore, in GBM, the inhibited expression of genes involved in the CD4 T cell immune response, either by strengthening the negative regulation of their proliferation or by reducing MHC class II antigen processing/presentation, represents an intrinsic feature that discriminates primary vs secondary brain tumors showing exactly opposite characteristics. The role of CD4 T cells in the rejection of solid tumors has been recently described. In fact, CD4 T cells can kill cancer cells, if they express MHC II, induce tumoricidal macrophages, destroy the tumor vasculature through cytokine release and help CD8 T cells in the effector phase [38, 39]. In addition, CD4 + T cells may interact directly with microglia, promoting IFN-γ-dependent microglia activation and phagocytosis, necessary for tumor suppression [40].

These findings shed light on how GBMs established an immune-suppressive environment and may explain the limited success of ICI therapy in GBM compared to MM-BM lesions. Different studies demonstrated that epigenetic mechanisms mediate reprogramming of glioma cells resulting in the modification of immune cells and induction of a pro-tumorigenic TME [16]. Thus, building upon the demonstrated immunomodulatory activity of DHAs in different tumor histotypes, including GBM [17, 22], we investigated whether the epigenetic remodeling of GBM cells induced by guadecitabine could shift them towards a more immune susceptible and ICI-responsive state, similar to what is observed in BM.

The highest number of DEGs significantly modulated by guadecitabine, observed in GBM vs BM cells, is in line with the high susceptibility of glioma to epigenetic remodeling. More specifically, immune pathways involved in the activation, proliferation and migration of T and B cells and in the antigen processing and presentation via MHC class II, constitutively down-regulated in GBM vs MM-BM, were among the most frequently activated biological processes in GBM, after guadecitabine treatment. These immunomodulatory effects are indicative of the role of epigenetic remodeling to bring GBM cells closer to MM-BM cells.

In a broader view, the possibility of using guadecitabine to modulate GBM phenotype making it more immunogenic and possibly more responsive to immunotherapy, is also demonstrated by the activation of a set of URs involved in immune recognition, enhancing cellular response to IFN signaling, further boosting the activation and proliferation of NK and NK T cells, critically involved in innate and adaptive immune response. This is of pivotal importance, given the involvement of IFN-γ in immune cell infiltration and immune checkpoint molecule expression, which are closely associated to the efficacy of immunotherapy and survival rate in patients with glioma [32].

Moreover, we observed that guadecitabine exerted a stronger activation of gene encoding IFN alpha/beta receptor (IFNAR) in GBM vs MM-BM and MM cell lines. Although IFNAR1 depletion has been recently described as associated with decreased motility and invasion of glioma cells, leading to improved survival rates [41], many studies demonstrated the role of this receptor in restoring the immunosuppressive microenvironment in GBM, driven by the constitutive absence or downregulation of IFNAR, and in facilitating the infiltration and activation of immune cells within the TME [42, 43]. In addition to those positively modulated by guadecitabine, several genes, critically involved in cell proliferation and cell cycle regulation, tissue homeostasis and metabolism (e.g., fatty acid homeostasis, lipoprotein metabolic process, regulation of cellular respiration) were inhibited in GBM by treatment. Fatty acids can act as critical bio-energetic substrates within the GBM cells supporting both respiratory and proliferative activity, thus leading to the association of inhibition of fatty acid synthesis with the reduction of glioma cells proliferation [44, 45]. Additionally, guadecitabine inhibited, mainly in GBM vs MM-BM and/or MM, several URs that enriched for Hippo signaling strongly involved in gliomas progression, in the activation of chemoresistance mechanisms and in the development of an immunosuppressive microenvironment [46].

The epigenetic remodeling induced by guadecitabine in the profile of GBM cells, involved also genes with a prognostic relevance. In fact, genes associated with reduced or high risk GBM were up-regulated or down-regulated by DHA, respectively. Specifically, among the DHA up-regulated genes, we found ZBTB6 that significantly suppressed migration, invasion, and proliferation in GBM [47, 48], as well as DAXX involved in the suppression of tumor growth and increase of GBM patients’ survival [49]. Similarly, DHA down-regulated genes potentially associated to a low OS rate and poor prognosis in GBM such as ERLIN2 and TMEM41B that can inhibit cancer cell growth and metastasis [50, 51]. Overall, data obtained indicate that guadecitabine treatment might have a significant role in modulating the profile of GBM cells, increasing their potential sensitivity to immunotherapy. This evidence lays the ground for novel DHA-based combined immunotherapeutic approaches to improve the treatment of GBM patients.

### Supplementary Information

**Additional file 1: Table S1. **Clinical characteristics of GBM patients. **Table S2.** Clinical characteristics of MM-BM patients. **Table S3.** Clinical characteristics of MM patients.

**Additional file 2:** **Table S1**: Expression of specific genes of Cluster 1; **Table S2**: Enrichment analysis of Cluster 1; **Table S3**: Expression of specific genes of Cluster 2; **Table S4**: Enrichment analysis of Cluster 2; **Table S5**: Expression of specific genes of Cluster 3; **Table S6**: Enrichment analysis of Cluster 3; **Table S7**: Expression of specific genes of Cluster 4; **Table S8**: Enrichment analysis of Cluster 4; **Table S9**: Expression of specific genes of Cluster 5; **Table S10**: Enrichment analysis of Cluster 5; **Table S11**: Expression of specific genes of Cluster 6; **Table S12**: Enrichment analysis of Cluster 6.

**Additional file 3:** **Table S1**: Expression of specific genes of MHC class II_activation signature; **Table S2**: Expression of specific genes of MHC class II_inhibition signature; **Table S3**: Expression of specific genes of EMT process_activation signature; **Table S4**: Expression of specific genes of Tsoi signature.

**Additional file 4:** **Table S1**: Validation of GBM gene expression.

**Additional file 5:** **Table S1**: TCGA dataset_prognostic significance in GBM; **Table S2**: TCGA top 10 genes with high and low risk in GBM.

**Additional file 6.** **Table S1**: Canonical pathways modulated by guadecitabine in all tumor types.

**Additional file 7:** **Table S1**: Upstream regulators by guadecitabine in all tumor types; **Table S2**: Enrichment analysis of Upstream regulators up-regulated in GBM vs MM-BM; **Table S3**: Enrichment analysis of Upstream regulators down-regulated in GBM vs MM-BM; **Table S4**: Enrichment analysis of Upstream regulators up-regulated in GBM vsMM; **Table S5**: Enrichment analysis of Upstream regulators down-regulated in GBM vs MM; **Table S6**: Enrichment analysis of Upstream regulators up-regulated in GBM and MM-BM; **Table S7**: Enrichment analysis of Upstream regulators up-regulated in GBM and MM; **Table S8**: Enrichment analysis of Upstream regulators down-regulated in GBM and MM-BM; **Table S9**: Enrichment analysis of Upstream regulators down-regulated in GBM and MM.

**Additional file 8: Figure S1.** Venn-diagrams of DEGs modulated by guadecitabine treatment in GBM, MM-BM and cell lines. Venn-diagrams illustrate the overlaps of statistically significant (p diagrams illustrate the overlaps of statistically significant (p diagrams illustrate the overlaps of statistically significant (p value < 0.05) DEGs (p value < 0.05). A up- B down-regulated by guadecitabine treatment in all investigated GBM, MM-BM and MM cell lines.

## Data Availability

All data analysed during this study are included in this published article as supplementary information files and/or are available from the corresponding author on reasonable request.

## Abbreviations

GBM: Glioblastoma multiforme
ICI: Immune-checkpoint inhibitors
BM: Brain metastases
OS: Overall survival
IFN-γ: Interferon-gamma
TME: Tumor microenvironment
BBB: Blood–brain barrier
MHC: Major histocompatibility complex
DHA: DNA hypomethylating agent
CTL: Cytotoxic T lymphocytes
CTA: Cancer testis antigens
MM: Melanoma
TCGA: The Cancer Genome Atlas
FBS: Fetal bovine serum
β: Beta values
DEG: Differentially expressed genes
IPA: Ingenuity pathway analysis
CP: Canonical pathways
UR: Upstream-regulators
GO: Gene Ontology
BP: Biological process
HR: Hazard ratio
EMT: Epithelial mesenchymal transition
IFNAR: IFN alpha/beta receptor
