# Supplementary material for: Epigenetic remodeling to improve the efficacy of immunotherapy in human glioblastoma: pre-clinical evidence for development of new immunotherapy approaches
Source: J Transl Med. 2024 Mar 1;22:223. doi: 10.1186/s12967-024-05040-x (PMC10908027; doi:10.1186/s12967-024-05040-x)
Supplement: Supplementary file 8 — Additional file 8: Figure S1. Venn-diagrams of DEGs modulated by guadecitabine treatment in GBM, MM-BM and cell lines. Venn-diagrams illustrate the overlaps of statistically significant (p diagrams illustrate the overlaps of statistically significant (p diagrams illustrate the overlaps of statistically significant (p value < 0.05) DEGs (p value < 0.05). A up- B down-regulated by guadecitabine treatment in all investigated GBM, MM-BM and MM cell lines. [file 12967_2024_5040_MOESM8_ESM.pdf]

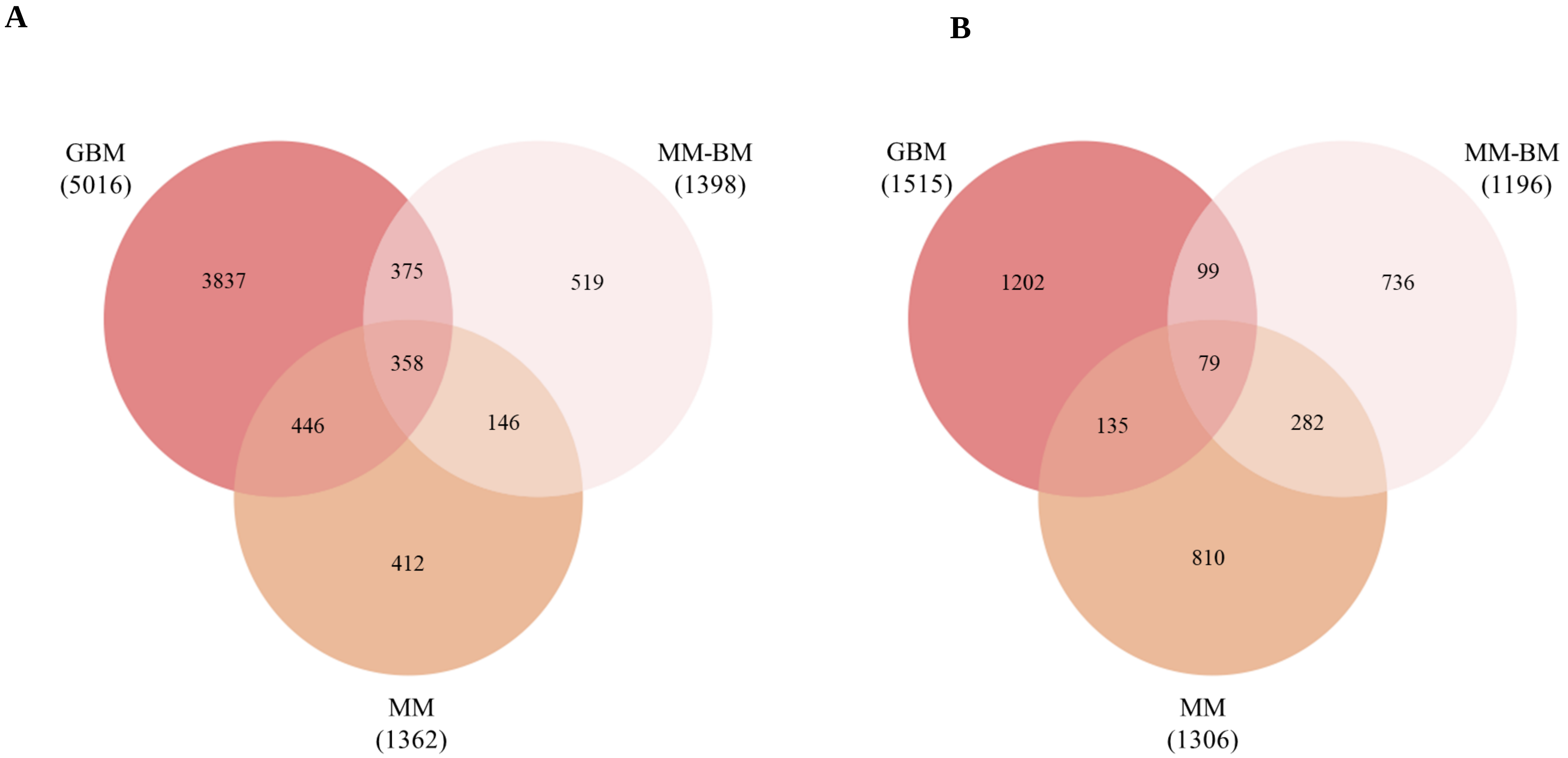

Additional Figure S1. Venn-diagrams of DEGs modulated by guadecitabine treatment in GBM, MM-BM and MM cell lines. Venn-diagrams illustrate the overlaps of the statistically significant (p-value<0.05) DEGs (p-value<0.05) **A** up- or **B** down-regulated by guadecitabine treatment in all investigated GBM, MM-BM and MM cell lines.
